# Supplementary material for: Astrocytic phagocytosis contributes to demyelination after focal cortical ischemia in mice
Source: Nat Commun. 2022 Mar 3;13:1134. doi: 10.1038/s41467-022-28777-9 (PMC8894352; doi:10.1038/s41467-022-28777-9)
Supplement: Supplementary file 1 — Supplementary Information [file 41467_2022_28777_MOESM1_ESM.pdf]

Supplementary Materials for

Astrocytic phagocytosis contributes to demyelination after focal cortical ischemia in mice

Ting Wan†, Wusheng Zhu†, Ying Zhao†, Xiaohao Zhang†, Ruidong Ye, Meng Zuo, Pengfei Xu, Zhenqian Huang, Chunni Zhang*, Yi Xie*, Xinfeng Liu*

†Contributed equally.

*Corresponding author. E-mail: xfliu2@vip.163.com (X.L.) or xy_307@126.com (Y.X.) or zchunni27@hotmail.com (C.Z.)

The file includes:

Supplementary Fig. 1 Reactive astrogliosis in the demyelinating CC after cortical ischemia.

Supplementary Fig. 2 The cellular specificity of LCN2 expression in the CNS.

Supplementary Fig. 3 LPS stimulation properly mimics the inflammatory environment in vivo following dMCAO.

Supplementary Fig. 4 Absence of *Lcn2* abolishes reactive astrogliosis both in vivo and in vitro.

Supplementary Fig. 5 The specificity of LCN2 antibodies.

Supplementary Fig. 6 Ablation of *Lcn2* attenuates myelin loss after cortical ischemia.

Supplementary Fig. 7 Re-expression of LCN2 in $Lcn2^{-/-}$ astrocytes.

Supplementary Fig. 8 The cellular specificity of LV-mediated LCN2 re-expression in $Lcn2^{-/-}$ mice.

Supplementary Fig. 9 Specific re-expression of astrocytic LCN2 destroyed myelin sheath microstructure in $Lcn2^{-/-}$ mice.

Supplementary Fig. 10 The cellular specificity of in vivo lentiviral transfection of control and *Lrp1*-RNAi.

Supplementary Fig. 11 Immunoblotting and quantifications for the expression of LCN2 and LRP1/p38 signaling in astrocytes transfected with LV-NC and LV-*Lrp1*-RNAi.

Supplementary Fig. 12 *Lrp1* silencing reverses cytosolic LCN2-induced myelin phagocytosis in $Lcn2^{-/-}$ astrocytes.

Supplementary Fig. 13 Schematic depicting the role of astrocytic phagocytosis towards secondary demyelination in a mouse model of cortical ischemic stroke.

Supplementary Table 1 Real-time quantitative PCR primers.

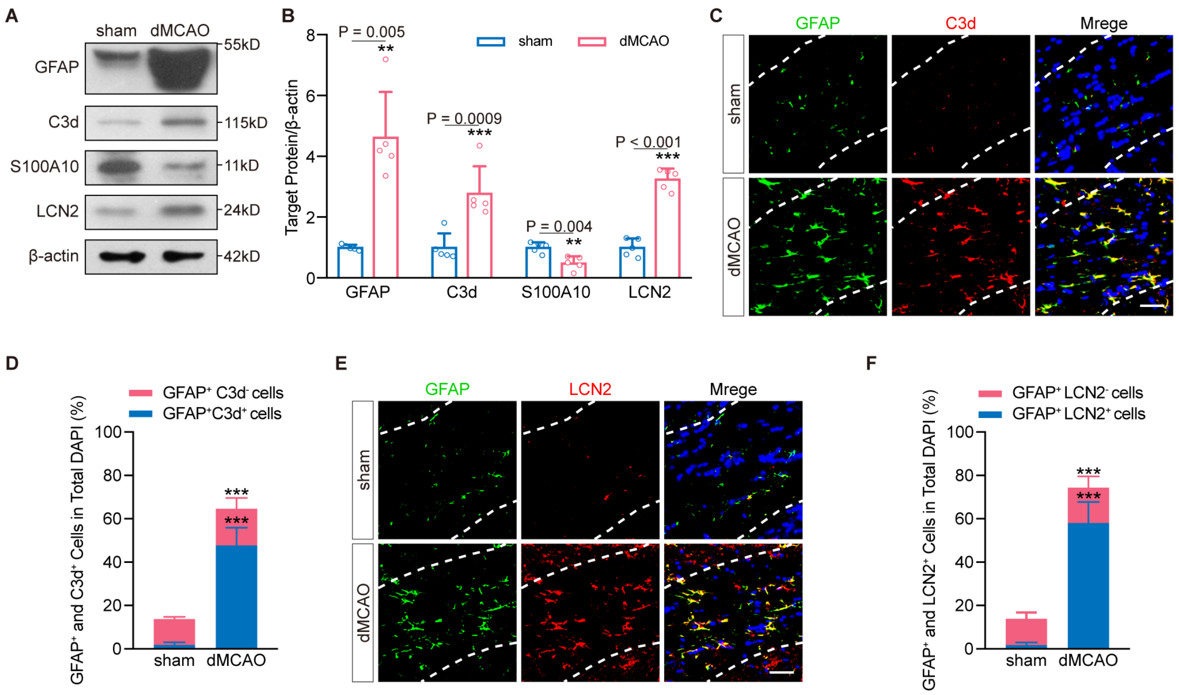

Supplementary Fig. 1 Reactive astrogliosis in the demyelinating CC after cortical ischemia. A, B Immunoblotting images and quantitative analyses of GFAP, C3d, S100A10 and LCN2 expressions (n = 5 mice; mean ± S.D.; **P < 0.01, ***P < 0.001 vs. sham; paired t-test). Protein samples derived from the same experiment and gels/blots were processed in parallel. **C, D** Immunofluorescent images and quantifications of C3d (red) expression in $GFAP^+$ (green) astrocytes 7 days after dMCAO (n = 5 mice; mean ± S.D.; compared to sham, ***P = 0.0008 for $GFAP^+$ astrocytes, ***P = 0.0003 for $GFAP^+$ $C3d^+$ astrocytes; paired t-test). Scale bar, 20 μm. **E, F** Double immunostaining and quantification displaying LCN2 (red) expression in $GFAP^+$ (green) astrocytes (n = 5 mice; mean ± S.D.; compared to sham, ***P = 0.0006 for $GFAP^+$ astrocytes, ***P = 0.0001 for $GFAP^+$ $LCN2^+$ astrocytes; paired t-test). Scale bar, 20 μm. Source data are provided as a Source Data file.

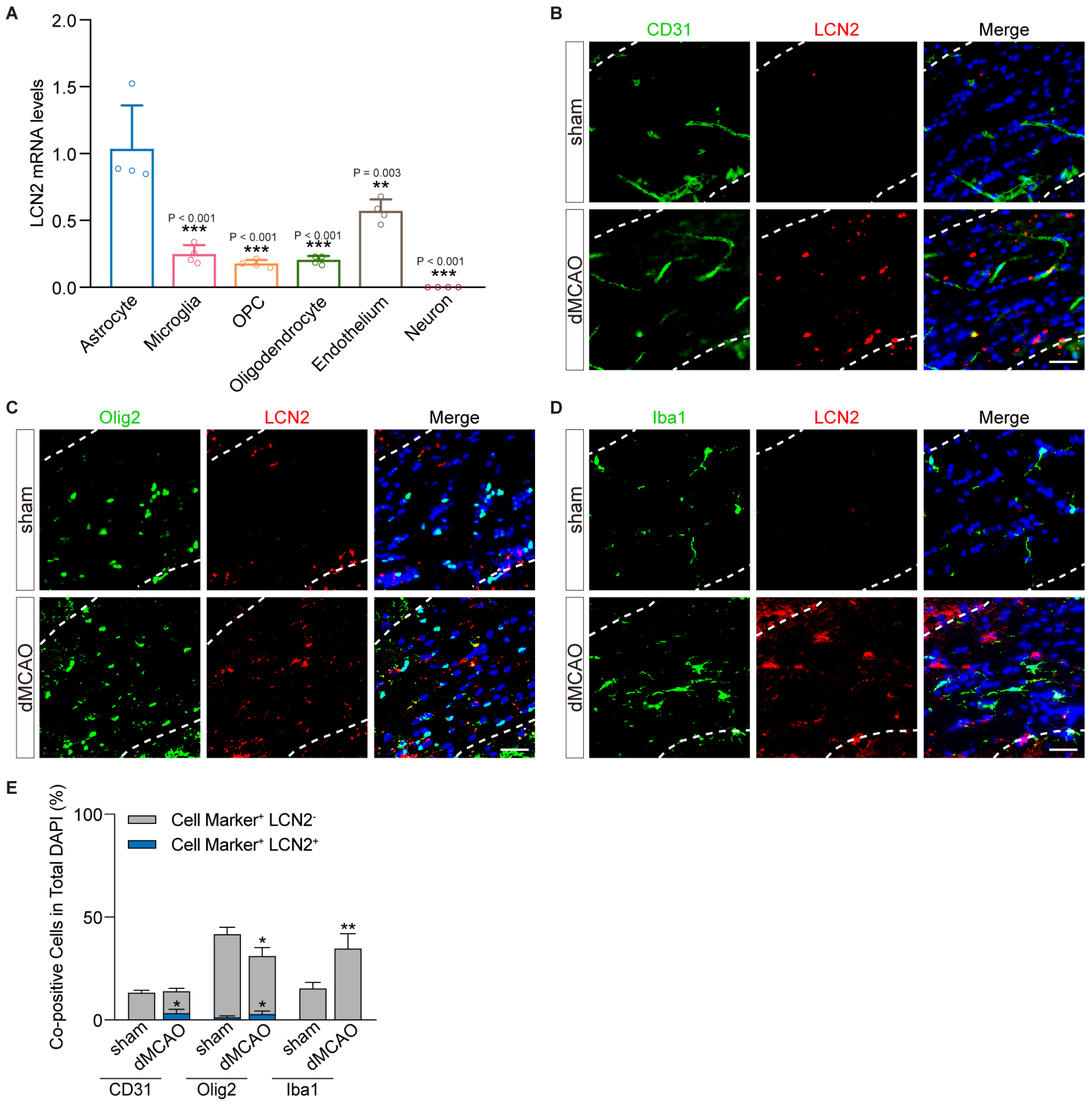

Supplementary Fig. 2 The cellular specificity of LCN2 expression in the CNS. A LCN2 mRNA level in astrocyte, microglia, OPC, oligodendrocyte, endothelium and neuron in vitro (n = 4 independent primary cell cultures; mean ± S.D.; **P < 0.01, ***P < 0.001 vs. astrocyte; one-way ANOVA, Tukey post hoc test). **B–D** Representative fluorescent images of LCN2 (red) with CD31 (endothelium marker, green), Olig2 (oligodendroglial lineage cell marker, green) and Iba1 (microglia marker, green) in vivo. Scale bar, 20 μm. **E** Quantification of co-positive cells after focal ischemia (n = 5 mice; mean ± S.D.; compared to sham, *P = 0.033 for Olig2^{+} cells, **P = 0.004 for Iba1^{+} cells, *P = 0.017 for CD31^{+} LCN2^{+} cells, *P = 0.047 for Olig2^{+} LCN2^{+} cells; paired t-test). Source data are provided as a Source Data file.

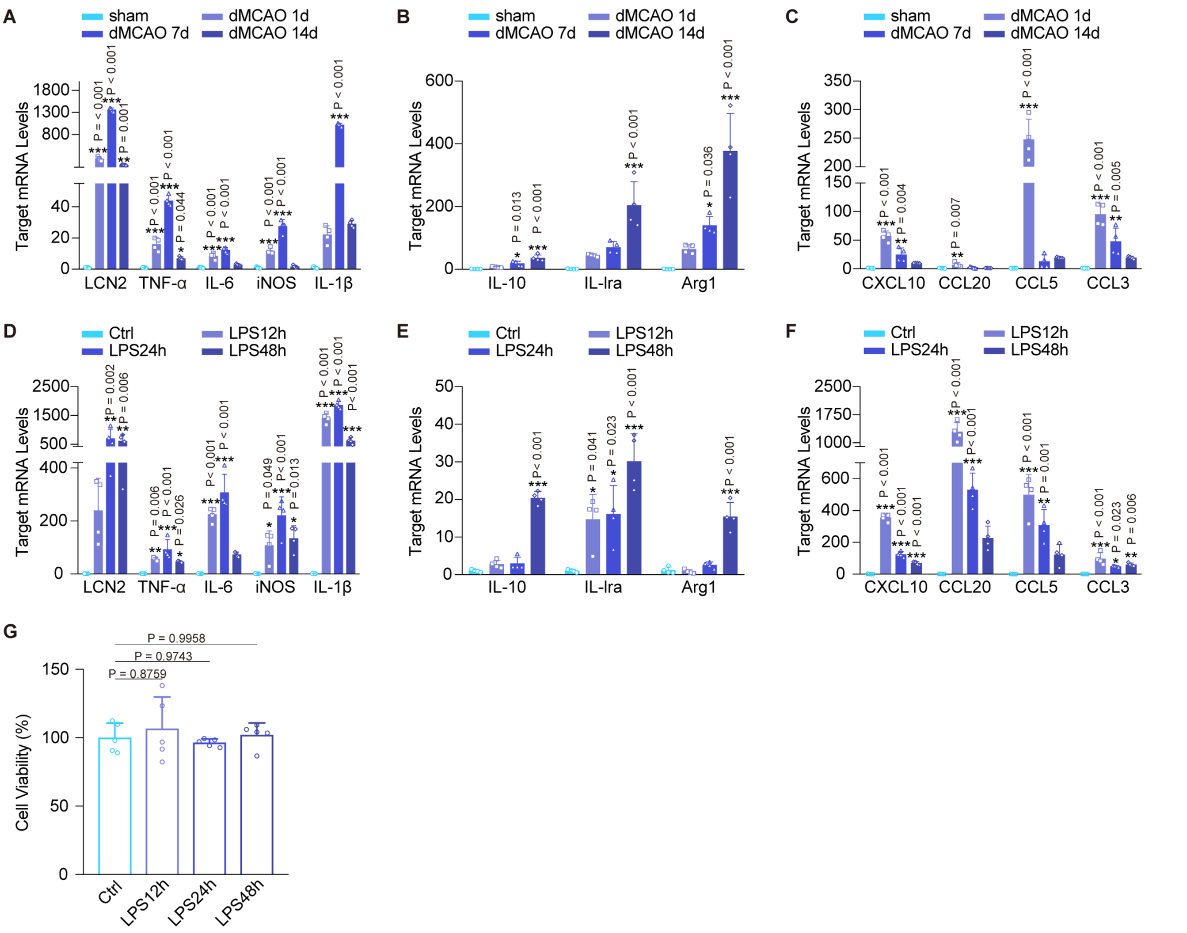

Supplementary Fig. 3 LPS stimulation properly mimics the inflammatory environment in vivo following dMCAO. A–C Relative mRNA expression levels of pro-inflammatory cytokines, anti-inflammatory cytokines, and chemokines in the ipsilateral CC after dMCAO at different time points (n = 4 mice; mean ± S.D.; *P < 0.05, **P < 0.01, ***P < 0.001 vs. sham; one-way ANOVA, Tukey post hoc test). **D–F** Relative mRNA expression levels of pro-inflammatory cytokines, anti-inflammatory cytokines and chemokines in primary astrocytes at different time points after LPS stimulation (n = 4 independent primary cell cultures; mean ± S.D.; *P < 0.05, **P < 0.01, ***P < 0.001 vs. Ctrl; one-way ANOVA, Tukey post hoc test). **G** Detection of cell viability with CCK-8 kit after LPS stimulation (n = 5 independent primary cell cultures; mean ± S.D.; one-way ANOVA, Tukey post hoc test). Source data are provided as a Source Data file.

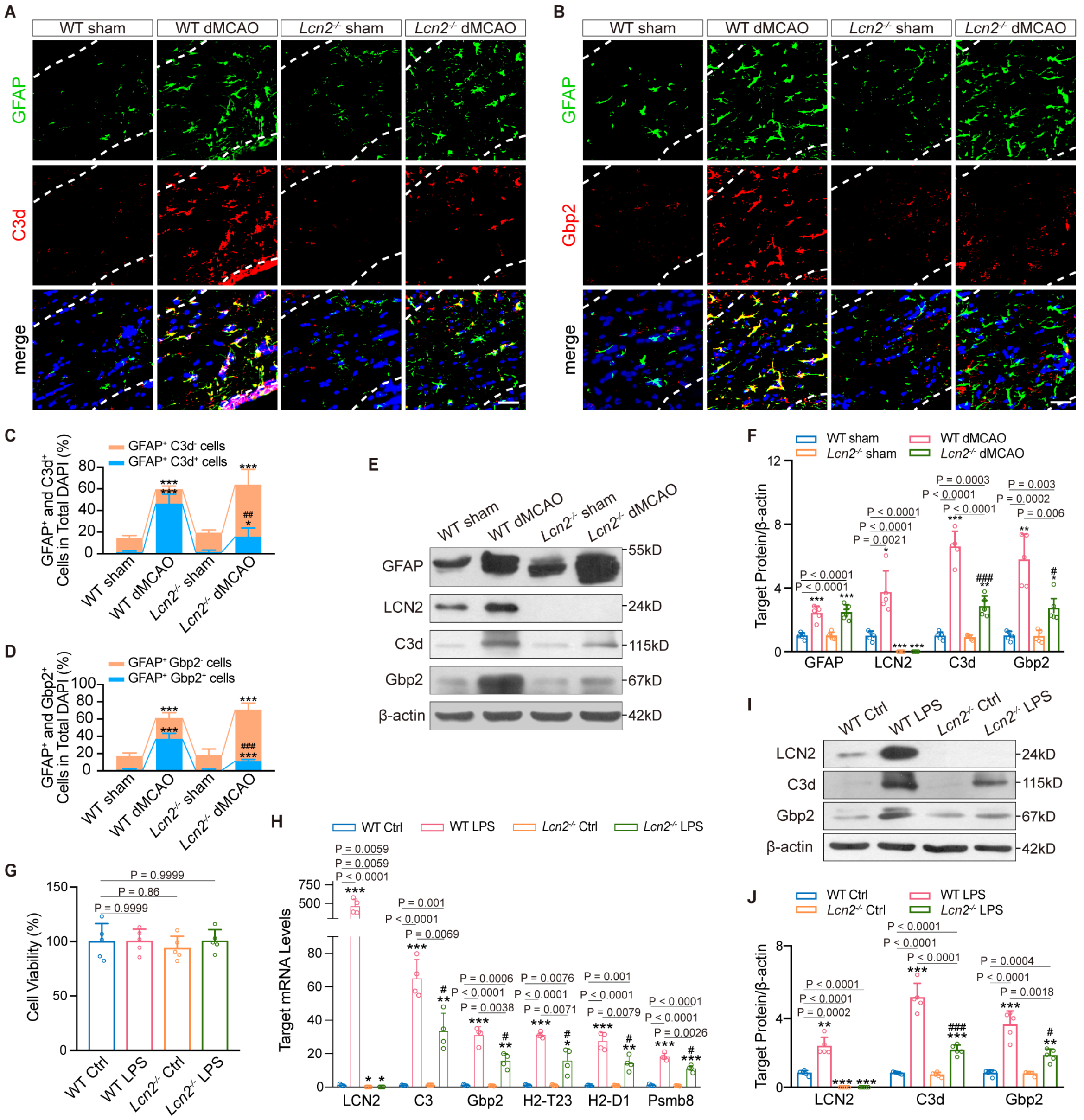

Supplementary Fig. 4 Absence of *Lcn2* abolishes reactive astrogliosis both in vivo and in vitro. A, B Immunofluorescent images of GFAP$^+$ (green) astrocyte and pro-inflammatory reactive markers (C3d$^+$ or Gbp2$^+$, red). Scale bar, 20 μm. **C, D** Quantification of the percentage of GFAP and reactive marker (C3d or Gbp2) co-positive cells (n = 5 mice; mean ± S.D.; two-way ANOVA, Tukey post hoc test or repeated-measures t-test; ***P < 0.001 vs. WT sham for Tukey post hoc test; adjusted *P < 0.0083, ***P < 0.0002 vs. WT sham; $^{\#\#}$P < 0.0017, $^{\#\#\#}$P < 0.0002 vs. WT dMCAO for repeated-measures t-test). **E, F** Immunoblotting analyses of GFAP, LCN2, C3d and Gbp2 expressions in WT and *Lcn2*$^{-/-}$ mice (n = 5 mice; mean ± S.D.; two-way ANOVA, Tukey post hoc test or repeated-measures t-test; ***P < 0.001 vs. WT sham for Tukey post hoc test; adjusted *P < 0.0083, **P < 0.0017, ***P < 0.0002 vs. WT sham; $^{\#}$P <

0.0083, $^{\#\#\#}P < 0.0002$ vs. WT dMCAO for repeated-measures t-test). **G** Detection of cell viability with CCK-8 kit after LPS stimulation (n = 5 independent primary cell cultures; mean ± S.D.; two-way ANOVA, Tukey post hoc test). **H** mRNA expression of astrocytic reactive markers in different groups (n = 4 independent primary cell cultures; mean ± S.D.; adjusted $^{*}P < 0.0083$, $^{**}P < 0.0017$, $^{***}P < 0.0002$ vs. WT Ctrl; $^{\#}P < 0.0083$ vs. WT LPS; two-way ANOVA, repeated-measures t-test). **I, J** Immunoblotting analyses of LCN2, C3d and Gbp2 expressions (n = 5 independent primary cell cultures; mean ± S.D.; adjusted $^{**}P < 0.0017$, $^{***}P < 0.0002$ vs. WT Ctrl; $^{\#}P < 0.0083$, $^{\#\#\#}P < 0.0002$ vs. WT LPS; two-way ANOVA, repeated-measures t-test). For immunoblotting experiments, protein samples derived from the same experiment and gels/blots were processed in parallel. Source data are provided as a Source Data file.

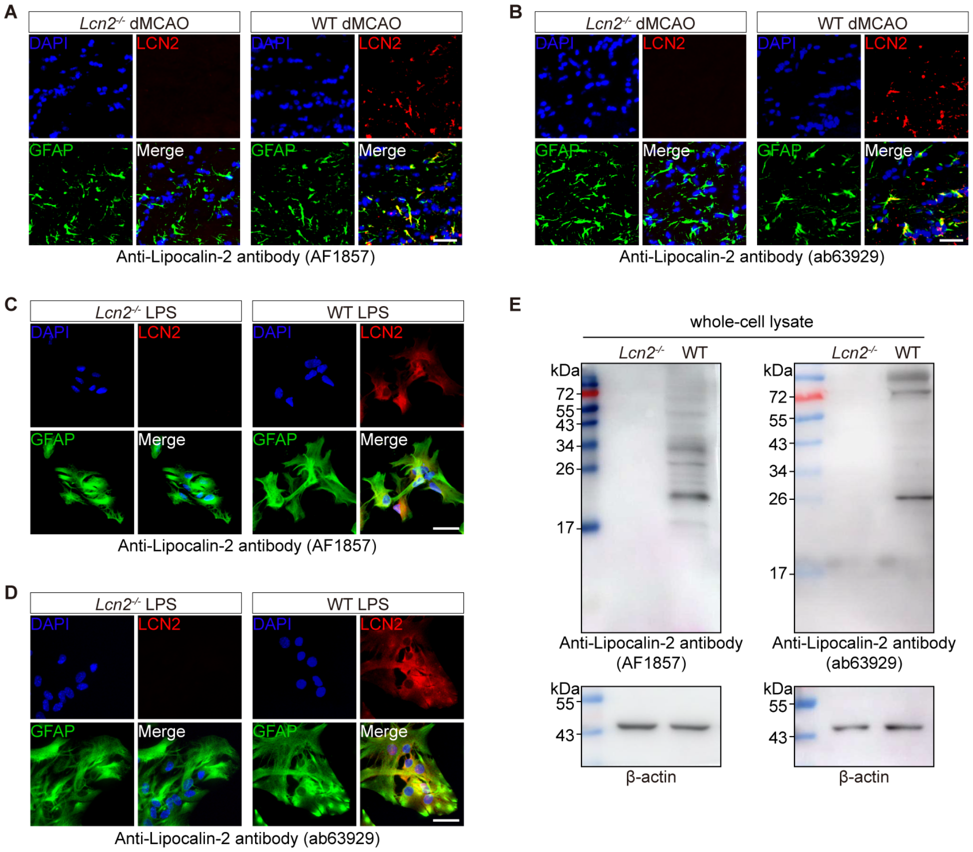

Supplementary Fig. 5 The specificity of LCN2 antibodies. A, B *Lcn2*$^{-/-}$ and WT dMCAO mice were stained with LCN2 (red; AF1857 from R&D, ab63929 from Abcam) and GFAP (green) antibodies (n = 5 mice). Scale bar, 20 μm. **C–E** Representative immunostaining and immunoblotting images from cultured primary *Lcn2*$^{-/-}$ and WT astrocytes showing the specificity of LCN2 antibody used in manuscript (n = 5 independent primary cell cultures). Scale bar, 20 μm. Protein samples derived from the same experiment and gels/blots were processed in parallel. Source data are provided as a Source Data file.

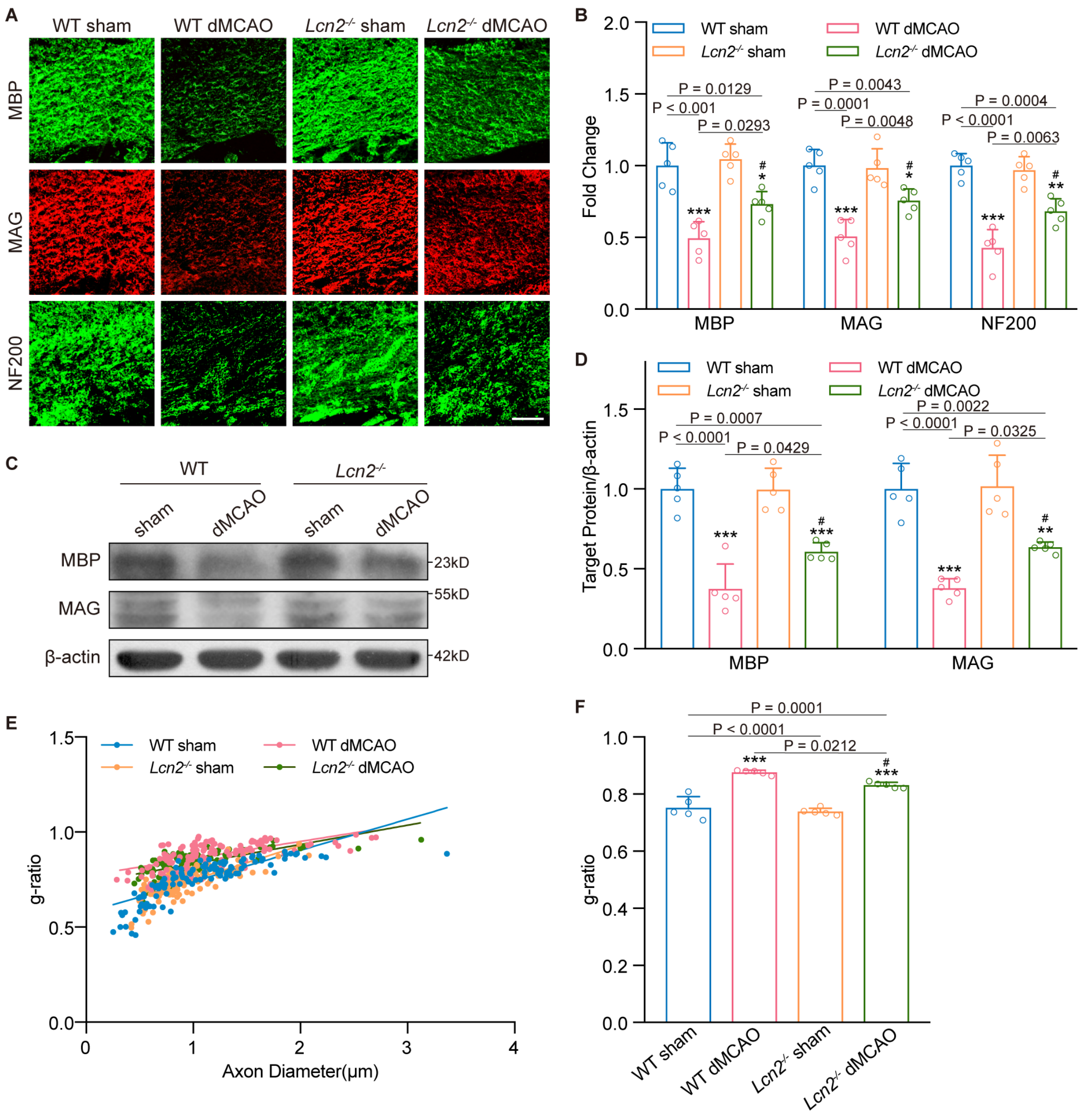

Supplementary Fig. 6 Ablation of *Lcn2* attenuates myelin loss after cortical ischemia. A, B MBP, MAG, NF200 immunostaining images and quantifications from WT and *Lcn2*$^{-/-}$ mice (n = 5 mice; mean ± S.D.; two-way ANOVA, Tukey post hoc test or repeated-measures t-test; *P < 0.05, ***P < 0.001 vs. WT sham; #P < 0.05 vs. WT dMCAO for Tukey post hoc test; adjusted *P < 0.0083, **P < 0.0017, ***P < 0.0002 vs. WT sham; #P < 0.0083 vs. WT dMCAO for repeated-measures t-test). Scale bar, 50 μm. **C, D** Immunoblotting and statistical analyses of the expression of MBP and MAG (n = 5 mice; mean ± S.D.; **P < 0.01, ***P < 0.001 vs. WT sham; #P < 0.05 vs. WT dMCAO; two-way ANOVA, Tukey post hoc test). Protein samples derived from the same experiment and gels/blots were processed in parallel. **E, F** Scatter plots and inset bar graph of the g-ratio from electron micrographs of myelin sheath (n = 5 mice; mean ± S.D.; ***P < 0.001 vs. WT sham; #P < 0.05 vs. WT dMCAO; two-way ANOVA, Tukey post hoc test). Source data are provided as a Source Data file.

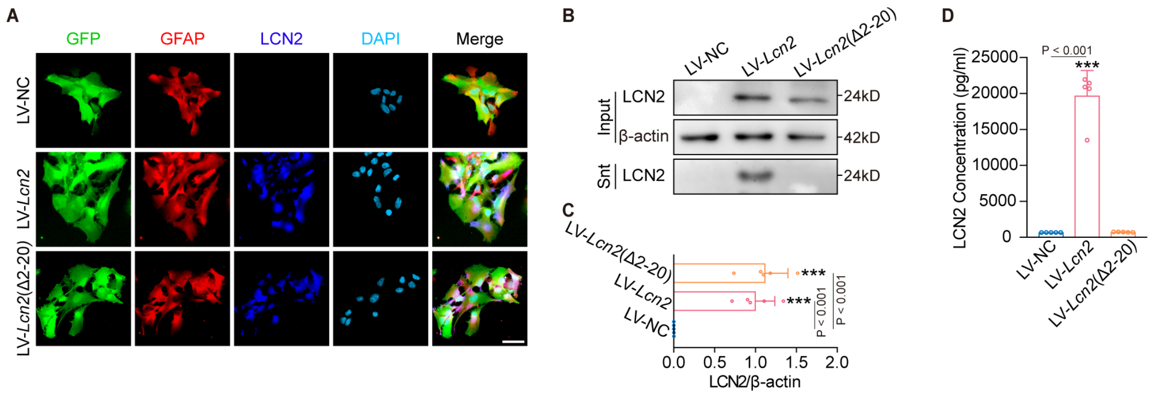

Supplementary Fig. 7 Re-expression of LCN2 in *Lcn2*$^{-/-}$ astrocytes. A Representative fluorescence images of GFP (green), GFAP (red), LCN2 (blue) and DAPI showing the re-expression of LCN2 after LV transfection (n = 5 independent primary cell cultures). Scale bar, 20 μm. **B** Representative immunoblotting images of LCN2 in whole cell extract (Input) and in extracellular media (supernatant, Snt). **C** Immunoblotting quantitative analysis of LCN2 expression in whole cell extract (n = 5 independent primary cell cultures; mean ± S.D.; ***P < 0.001 vs. LV-NC; one-way ANOVA, Tukey post hoc test). Protein samples derived from the same experiment and gels/blots were processed in parallel. **D** ELISA detection of supernatant LCN2 (n = 5 independent extracellular medium; mean ± S.D.; ***P < 0.001 vs. LV-NC; one-way ANOVA, Tukey post hoc test). Source data are provided as a Source Data file.

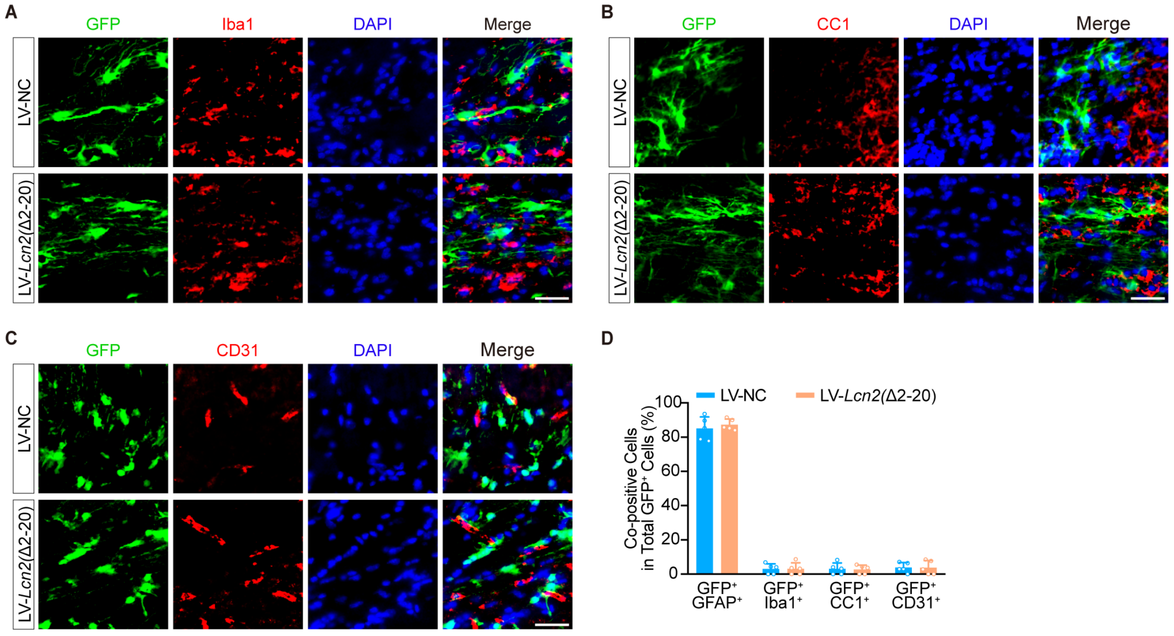

Supplementary Fig. 8 The cellular specificity of LV-mediated LCN2 re-expression in *Lcn2*$^{-/-}$ mice. A–C Doble-staining of GFP (green) with Iba1 (microglia marker, red), CC1 (oligodendrocyte marker, red) or CD31 (endothelium marker, red) were performed in LV-NC and LV-*Lcn2*(Δ2–20) mice. Scale bar, 20 μm. **D** Quantification of the percentage of co-positive cells in total GFP^{+} cells (n = 5 mice; mean ± S.D.; paired t-test). Source data are provided as a Source Data file.

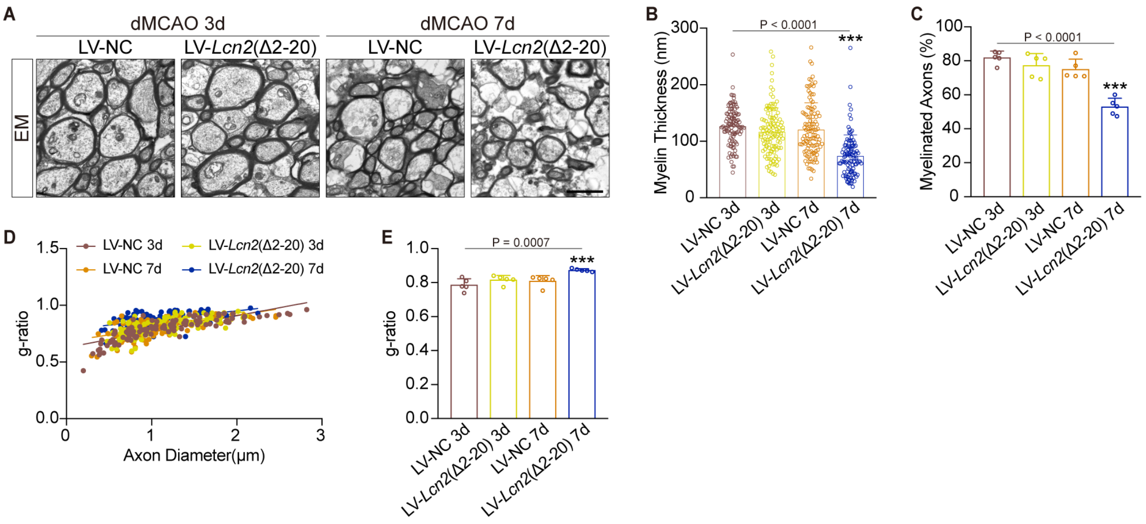

Supplementary Fig. 9 Specific re-expression of astrocytic LCN2 destroyed myelin sheath microstructure in *Lcn2*$^{-/-}$ mice. A Representative EM images in *Lcn2*$^{-/-}$ mice transfected with LV-NC or LV-*Lcn2*(Δ2–20). Scale bar, 2 μm. **B–E** Statistical analyses of myelin thickness, myelinated axons and g-ratio (n = 5 mice; mean ± SD; two-way ANOVA, Tukey post hoc test or repeated-measures t-test; ***P < 0.001 vs. LV-NC 3d for Tukey post hoc test; adjusted ***P < 0.0002 vs. LV-NC 3d for repeated-measures t-test). Source data are provided as a Source Data file.

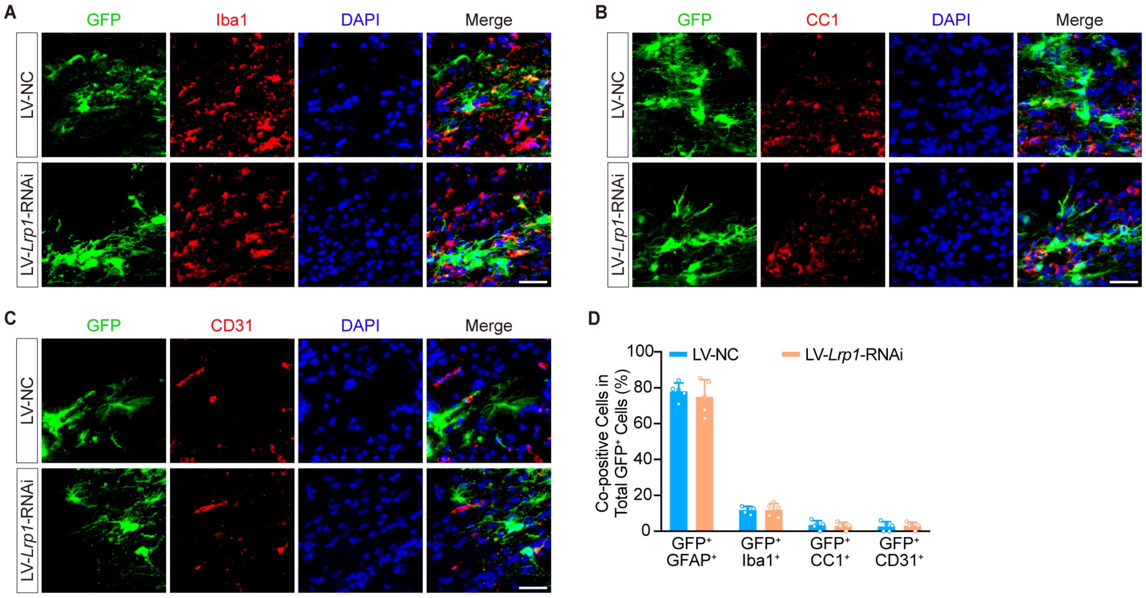

Supplementary Fig. 10 The cellular specificity of in vivo lentiviral transfection of control and *Lrp1*-RNAi. A–C Representative fluorescence images of the transfection of lentivirus into microglia (Iba1$^+$, red), mature oligodendrocytes (CC1$^+$, red) and vascular endothelial cells (CD31$^+$, red). Scale bar, 20 μm. **D** Quantification of the percentage of co-positive cells in total GFP$^+$ cells (n = 5 mice; mean ± S.D.; paired t-test). Source data are provided as a Source Data file.

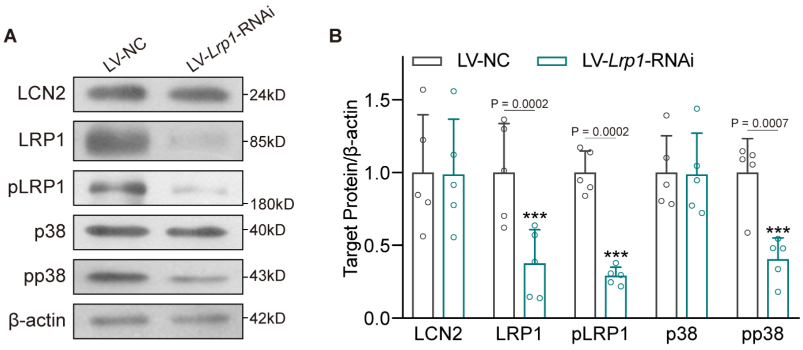

Supplementary Fig. 11 Immunoblotting and quantifications for the expression of LCN2 and LRP1/p38 signaling in astrocytes transfected with LV-NC and LV-*Lrp1*-RNAi. A Representative immunoblotting images of LCN2 and LRP1/p38 signaling (LRP1, pLRP1, p38 and pp38) after lentivirus transfection. **B** Immunoblotting analyses of LCN2, LRP1, pLRP1, p38 and pp38 expressions (n = 5 independent primary cell cultures; mean ± S.D.; ***P < 0.001 vs. LC-NC; paired t-test). Protein samples derived from the same experiment and gels/blots were processed in parallel. Source data are provided as a Source Data file.

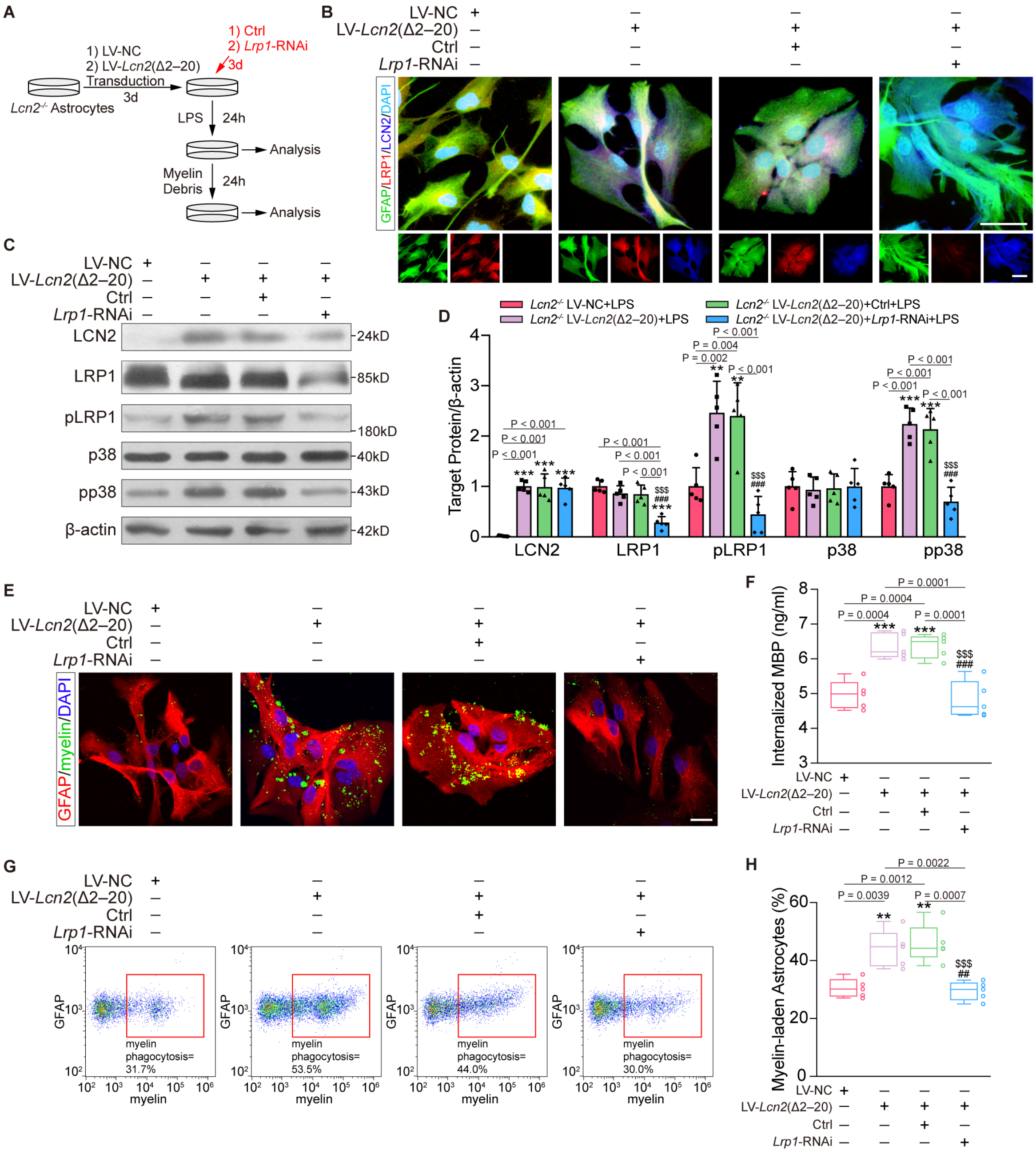

Supplementary Fig. 12 ***Lrp1*** **silencing reverses cytosolic LCN2-induced myelin phagocytosis in** ***Lcn2***$^{-/-}$ **astrocytes. A** Experimental design for primary *Lcn2*$^{-/-}$ astrocytes. **B** Representative immunocytochemistry images of GFAP (green), LRP1 (red), LCN2 (blue) and DAPI (cyan) showing positive staining of LCN2 after transfection of LV-*Lcn2*(Δ2–20) and decreased staining of LRP1 after supplementation of *Lrp1*-RNAi plasmid. Scale bar, 20 μm. **C, D** Immunoblotting demonstrations and analyses of LCN2 and LRP1/p38 pathway (n = 5 independent primary cell cultures; mean ± S.D.; $^{**}P < 0.01$, $^{***}P < 0.001$ vs. *Lcn2*$^{-/-}$ LV-NC+LPS; $^{\#\#\#}P < 0.001$ vs. *Lcn2*$^{-/-}$ LV-*Lcn2*(Δ2–20)+LPS; $^{\$\$\$}P < 0.001$ vs. *Lcn2*$^{-/-}$ LV-*Lcn2*(Δ2–20)+Ctrl+LPS; one-way ANOVA, Tukey post hoc test). Protein samples derived from the same experiment and

gels/blots were processed in parallel. **E–H** Representative confocal images, ELISA and flow cytometry analyses showing the differences of astrocytic phagocytosis (n = 5 independent primary cell cultures; mean ± S.D.; $^{**}P < 0.01$, $^{***}P < 0.001$ vs. *Lcn2*$^{-/-}$ LV-NC+LPS; $^{\#\#}P < 0.01$, $^{\#\#\#}P < 0.001$ vs. *Lcn2*$^{-/-}$ LV-*Lcn2*(Δ2–20)+LPS; $^{\$\$\$}P < 0.001$ vs. *Lcn2*$^{-/-}$ LV-*Lcn2*(Δ2–20)+Ctrl+LPS; one-way ANOVA, Tukey post hoc test). Scale bar, 20 μm. In the box plots (**F, H**), the middle bar represents the median, the box represents the interquartile range, and whiskers indicate the maximum and minimum values. Dots are all the data points. Source data are provided as a Source Data file.

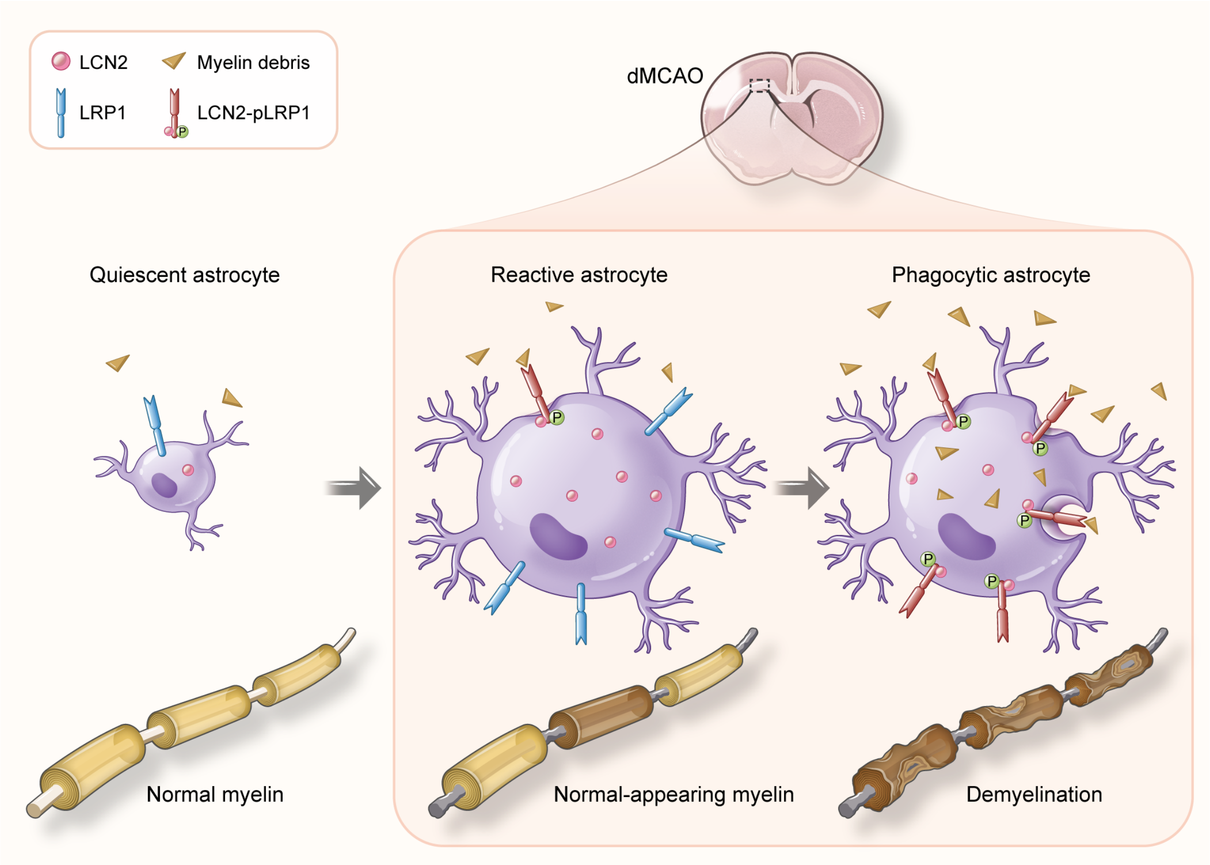

Supplementary Fig. 13 Schematic depicting the role of astrocytic phagocytosis towards secondary demyelination in a mouse model of cortical ischemic stroke. LCN2/LRP1 signaling participates in astrocyte-mediated myelin phagocytosis and contributes to secondary demyelination in nonischemic corpus callosum after focal cortical ischemia.

Supplementary Table 1 Real-time quantitative PCR primers.

Primer	Forward primer (5-3')	Reverse primer (3-5')
Arg1	CTCCAAGCCAAAGTCCTTAGAG	GGAGCTGTCATTAGGGACATCA
C3	TCCAACAAGAACACCCTCA	GGCTGGATAAGTCCCACA
CCL20	ACTGTTGCCTCTCGTACATACA	GAGGAGGTTCACAGCCCTTTT
CCL3	TGTACCATGACACTCTGCAAC	CAACGATGAATTGGCGTGGAA
CCL5	TTTGCCTACCTCTCCCTCG	CGACTGCAAGATTGGAGCACT
CXCL10	CCAAGTGCTGCCGTCATTTTC	GGCTCGCAGGGATGATTTCAA
Gbp2	GTGCCTCACCCCAAGAA	AGCAAAGATCCAGCAGTCA
H2-D1	TCCCTTCCATAGCCAACC	TCCAGCCACCCACATTC
H2-T23	TCAGAGATGGTCGATGAGG	ATGTGCCTTTGGAGGGT
IL-10	CTTACTGACTGGCATGAGGATCA	GCAGCTCTAGGAGCATGTGG
IL-1β	GAAATGCCACCTTTTGACAGTG	TGGATGCTCTCATCAGGACAG
IL-6	CTGCAAGAGACTTCCATCCAG	AGTGGTATAGACAGGTCTGTTGG
IL-lra	GCTCATTGCTGGGTACTTACAA	CCAGACTTGGCACAAGACAGG
iNOS	ACATCGACCCGTCCACAGTAT	CAGAGGGGTAGGCTTGTCTC
LCN2	TGGCCCTGAGTGTCATGTG	CTCTTGTAGCTCATAGATGGTGC
Psmb8	ATGCCTATGGGGTGATGG	CCGTCTTCCTTCATGTGGT
TNF-α	CAGGCGGTGCCTATGTCTC	CGATCACCCCGAAGTTCAGTAG
GAPDH	AAGAAGGTGGTGAAGCAGG	GAAGGTGGAAGAGTGGGAGT
