# Supplementary material for: Astrocytic phagocytosis contributes to demyelination after focal cortical ischemia in mice
Source: Nat Commun. 2022 Mar 3;13:1134. doi: 10.1038/s41467-022-28777-9 (PMC8894352; doi:10.1038/s41467-022-28777-9)
Supplement: Supplementary file 2 — Reporting Summary [file 41467_2022_28777_MOESM2_ESM.pdf]

nature portfolio

Corresponding author(s): Yi Xie

Last updated by author(s): Jan 31, 2022

Reporting Summary

Nature Portfolio wishes to improve the reproducibility of the work that we publish. This form provides structure for consistency and transparency in reporting. For further information on Nature Portfolio policies, see our Editorial Policies and the Editorial Policy Checklist.

Statistics

For all statistical analyses, confirm that the following items are present in the figure legend, table legend, main text, or Methods section.

n/a	Confirmed	
☐	☒	The exact sample size (*n*) for each experimental group/condition, given as a discrete number and unit of measurement
☐	☒	A statement on whether measurements were taken from distinct samples or whether the same sample was measured repeatedly
☐	☒	The statistical test(s) used AND whether they are one- or two-sided<br>*Only common tests should be described solely by name; describe more complex techniques in the Methods section.*
☐	☒	A description of all covariates tested
☐	☒	A description of any assumptions or corrections, such as tests of normality and adjustment for multiple comparisons
☐	☒	A full description of the statistical parameters including central tendency (e.g. means) or other basic estimates (e.g. regression coefficient) AND variation (e.g. standard deviation) or associated estimates of uncertainty (e.g. confidence intervals)
☐	☒	For null hypothesis testing, the test statistic (e.g. *F*, *t*, *r*) with confidence intervals, effect sizes, degrees of freedom and *P* value noted<br>*Give P values as exact values whenever suitable.*
☒	☐	For Bayesian analysis, information on the choice of priors and Markov chain Monte Carlo settings
☒	☐	For hierarchical and complex designs, identification of the appropriate level for tests and full reporting of outcomes
☒	☐	Estimates of effect sizes (e.g. Cohen's *d*, Pearson's *r*), indicating how they were calculated

Our web collection on statistics for biologists contains articles on many of the points above.

Software and code

Policy information about availability of computer code

Data collection	FluoView FV3000; LSM800 confocal microscope; Olympus BX51 microscope; 7.0-T MRI scanner; Laser Speckle Contrast Imaging/LSCI (RWD Life Science CO.,LTD, China); microplate reader (Bio Tek, USA); Stratagene Mx3000P QPCR system (Agilent Technologies, USA); H7500 Transmission Electron Microscope (Hitachi, Japan); FACS Canto flow cytometer (Becton Dickinson)
Data analysis	FlowJo (version 10.4.0); ImageJ software (version 2.0.0); Imaris software (version 9.2.1); GraphPad Prism software (version 8.0); Adobe photoshop (version 21.0.2); ANY-maze video tracking software (Stoelting, USA).

For manuscripts utilizing custom algorithms or software that are central to the research but not yet described in published literature, software must be made available to editors and reviewers. We strongly encourage code deposition in a community repository (e.g. GitHub). See the Nature Portfolio guidelines for submitting code & software for further information.

Data

Policy information about availability of data

All manuscripts must include a data availability statement. This statement should provide the following information, where applicable:

- Accession codes, unique identifiers, or web links for publicly available datasets
- A description of any restrictions on data availability
- For clinical datasets or third party data, please ensure that the statement adheres to our policy

All relevant data are available within the manuscript and the supplementary materials. Source data are provided with this paper.

Field-specific reporting

Please select the one below that is the best fit for your research. If you are not sure, read the appropriate sections before making your selection.

- [x] Life sciences
- [ ] Behavioural & social sciences
- [ ] Ecological, evolutionary & environmental sciences

For a reference copy of the document with all sections, see nature.com/documents/nr-reporting-summary-flat.pdf

Life sciences study design

All studies must disclose on these points even when the disclosure is negative.

Sample size	No statistical method was utilized to calculate the sample size. The sample size was determined based on the literature and our previous studies (PMID: 28642575; PMID: 33141760; PMID: 30014904; PMID: 32959911; PMID: 30664769).
Data exclusions	No data were excluded from our analyses.
Replication	Each animal experiment was carried out at least three times unless otherwise indicated. For in vitro treatment, each condition was performed at least four times unless otherwise indicated. All attempts at replication were successful.
Randomization	Mice were randomly tagged and assigned into different groups using an iOS App “Random Tools (version 3.4.1)”. Randomization was stratified by cage and genotype to make sure that unknown covariates were controlled.
Blinding	Three of our co-authors, who were blinded to grouping, analyzed all the results of the study.

Reporting for specific materials, systems and methods

We require information from authors about some types of materials, experimental systems and methods used in many studies. Here, indicate whether each material, system or method listed is relevant to your study. If you are not sure if a list item applies to your research, read the appropriate section before selecting a response.

Materials & experimental systems

n/a	Involved in the study	
	x	Antibodies
x		Eukaryotic cell lines
x		Palaeontology and archaeology
	x	Animals and other organisms
x		Human research participants
x		Clinical data
x		Dual use research of concern

Methods

n/a	Involved in the study	
x		ChIP-seq
	x	Flow cytometry
	x	MRI-based neuroimaging

Antibodies

Antibodies used

CC1(1:400 for IF; OP80, Millipore)
CD31 (1:50 for IF; 550274,BD Biosciences)
Gbp2 (1:200 for IF; ab203238, Abcam)
Gbp2 (1:1,000 for WB; sc-166960, Santa Cruz Biotechnology)
GFAP (1:1,000 for IF; 1:5,000 for WB; ab7260, Abcam)
GFAP (1:500 for IF; 3670S, Cell Signaling Technology)
LAMP1 (5 μg/mL for IF; AF4320, R&D)
LCN2 (1:200 for IF; 1:800 for WB; ab63929, Abcam)
LCN2 (5 μg/mL for IF; AF1857, R&D)
LRP1 (1:100 for IF; 1:5,000 for WB; ab92544, Abcam)
LRP1 (1:50 for IF; sc-57351, Santa Cruz Biotechnology)
MAG (1:200 for IF; 1:1,000 for WB; ab89780, Abcam)
MBP (1:200 for IF; 1:1,000 for WB; ab40390, Abcam)
NF200 (1:200 for IF; ab7795, Abcam)
PLP (1:200 for IF; ab28486, Abcam)
S100A10 (1:5,000 for WB; ab76472, Abcam)
GFP (1:100 for IF; 2955S, Cell Signaling Technology)
GFP (1:100 for IF; 2956S, Cell Signaling Technology)
p38 (1:1,000 for WB; 9212S, Cell Signaling Technology)
pp38 (1:1,000 for WB; 4511S, Cell Signaling Technology)

β-actin (1:5,000 for WB; 8457S, Cell Signaling Technology)
dMBP (1:2,000 for IF; AB5864, Millipore)
Olig2 (1:500 for IF; AB9610, Millipore)
C3d (5 μg/mL for IF; 0.1 μg/mL for WB; AF2655, R&D)
Galectin-3 (5 μg/mL for IF; AF1197, R&D)
pLRP1 (1:1,000 for WB; PA5-101013, Thermo Fisher)
Iba1 (1:100 for IF; 019-19741, Wako)

Alexa Fluor® 488 AffiniPure Donkey Anti-Goat IgG (H+L) (1:200 for IF; 705-545-003, Jackson)
Alexa Fluor® 594 AffiniPure Donkey Anti-Goat IgG (H+L) (1:200 for IF; 705-585-003, Jackson)
Alexa Fluor® 647 AffiniPure Donkey Anti-Goat IgG (H+L) (1:200 for IF; 705-605-003, Jackson)
Alexa Fluor® 488 AffiniPure Donkey Anti-Rabbit IgG (H+L) (1:200 for IF; 711-545-152, Jackson)
Alexa Fluor® 594 AffiniPure Donkey Anti-Rabbit IgG (H+L) (1:200 for IF; 711-585-152, Jackson)
Alexa Fluor® 647 AffiniPure Donkey Anti-Rabbit IgG (H+L) (1:200 for IF; 711-605-152, Jackson)
Alexa Fluor® 488 AffiniPure Donkey Anti-Mouse IgG (H+L)(1:200 for IF; 715-545-150, Jackson)
Alexa Fluor® 594 AffiniPure Donkey Anti-Mouse IgG (H+L)(1:200 for IF; 715-585-150, Jackson)
Alexa Fluor® 488 AffiniPure Goat Anti-Rat IgG (H+L) (1:200 for IF; 112-545-003, Jackson)
Alexa Fluor® 594 AffiniPure Goat Anti-Rat IgG (H+L) (1:200 for IF; 112-585-003, Jackson)

Anti-mouse IgG, HRP-linked Antibody #7076 (1:8000 for WB; 7076S, Cell Signaling Technology)
Anti-rabbit IgG, HRP-linked Antibody #7074 (1:8000 for WB; 7074S, Cell Signaling Technology)
mouse anti-goat IgG-HRP: sc-2354 (1:8000 for WB;sc-2354, Santa Cruz Biotechnology)

Validation

The antibodies used in the study were all purchased from reputable commercial sources. All antibodies are widely used and validated by the providers or previous publications. Below are the manufacturer's links to the antibody information and relevant citations.

CC1(OP80, Millipore)(https://www.merckmillipore.com/CN/zh/product/Anti-APC-Ab-7-Mouse-mAb-CC-1,EMD_BIO-OP80?ReferrerURL=https%3A%2F%2Fcn.bing.com%2F&bd=1);
CD31 (550274,BD Biosciences) (https://www.bdbiosciences.com/zh-cn/products/reagents/flow-cytometry-reagents/research-reagents/single-color-antibodies-ruo/purified-rat-anti-mouse-cd31.550274);
Gbp2 (ab203238, Abcam)(https://www.abcam.cn/gbp2-antibody-ab203238.html);
Gbp2 (sc-166960, Santa Cruz Biotechnology)(https://www.scbt.com/p/gbp1-5-antibody-g-12?requestFrom=search);
GFAP (ab53554, Abcam)(https://www.abcam.cn/gfap-antibody-ab53554.html);
GFAP (ab7260, Abcam)(https://www.abcam.cn/gfap-antibody-ab7260.html);
GFAP (3670S, Cell Signaling Technology)(https://www.cellsignal.cn/products/primary-antibodies/gfap-ga5-mouse-mab/3670?site-search-type=Products&N=4294956287&Ntt=3670s&fromPage=plp&_requestid=947998);
LAMP1 (AF4320, R&D)(https://www.rndsystems.com/cn/products/mouse-lamp-1-cd107a-lumenal-domain-antibody_af4320);
LCN2 (ab63929, Abcam)(https://www.abcam.cn/lipocalin-2--ngal-antibody-ab63929.html);
LCN2 (AF1857, R&D)(https://www.rndsystems.com/cn/products/mouse-lipocalin-2-ngal-antibody_af1857);
LRP1 (ab92544, Abcam)(https://www.abcam.cn/lrp1-antibody-epr3724-ab92544.html);
LRP1 (sc-57351, Santa Cruz Biotechnology)(https://www.scbt.com/p/lrp1-antibody-5a6?requestFrom=search);
MAG (ab89780, Abcam)(https://www.abcam.cn/maggma-antibody-3c7-bsa-and-azide-free-ab89780.html);
MBP (ab40390, Abcam)(https://www.abcam.cn/myelin-basic-protein-antibody-ab40390.html);
NF200 (ab7795, Abcam)(https://www.abcam.cn/neurofilament-heavy-polypeptide-antibody-nf-01-ab7795.html);
PLP (ab28486, Abcam)(https://www.abcam.cn/myelin-plp-antibody-ab28486.html);
S100A10 (ab76472, Abcam)(https://www.abcam.cn/s100a10-antibody-epr3317-ab76472.html);
GFP (2955S, Cell Signaling Technology)(https://www.cellsignal.cn/products/primary-antibodies/gfp-4b10-mouse-mab/2955?site-search-type=Products&N=4294956287&Ntt=2955s&fromPage=plp&_requestid=948661);
GFP (2956S, Cell Signaling Technology)(https://www.cellsignal.cn/products/primary-antibodies/gfp-d5-1-rabbit-mab/2956?site-search-type=Products&N=4294956287&Ntt=2956s&fromPage=plp&_requestid=948730);
p38 (9212S, Cell Signaling Technology)(https://www.cellsignal.cn/products/primary-antibodies/p38-mapk-antibody/9212?site-search-type=Products&N=4294956287&Ntt=9212s&fromPage=plp&_requestid=948850);
pp38 (4511S, Cell Signaling Technology)(https://www.cellsignal.cn/products/primary-antibodies/phospho-p38-mapk-thr180-tyr182-d3f9-xp-rabbit-mab/4511?site-search-type=Products&N=4294956287&Ntt=4511s&fromPage=plp&_requestid=948894);
β-actin (8457S, Cell Signaling Technology)(https://www.cellsignal.cn/products/primary-antibodies/b-actin-d6a8-rabbit-mab/8457?site-search-type=Products&N=4294956287&Ntt=8457s&fromPage=plp&_requestid=948925);
dMBP (AB5864, Millipore)(https://www.sigmaaldrich.cn/CN/zh/product/mm/ab5864?context=product);
Olig2 (AB9610, Millipore)(https://www.sigmaaldrich.cn/CN/zh/product/mm/ab9610?context=product);
C3d (0AF2655, R&D)(https://www.rndsystems.com/cn/products/mouse-rat-complement-component-c3d-antibody_af2655);
Galectin-3 (AF1197, R&D)(https://www.rndsystems.com/cn/products/human-mouse-rat-galectin-3-antibody_af1197);
pLRP1 (Thermo Fisher)(https://www.thermofisher.cn/cn/zh/antibody/product/Phospho-LRP1-Ser4520-Antibody-Polyclonal/PA5-101013);
Iba1 (019-19741, Wako)(https://labchem-wako.fujifilm.com/us/product/detail/W01W0101-1974.html).

Animals and other organisms

Policy information about studies involving animals; ARRIVE guidelines recommended for reporting animal research

Laboratory animals

Male C57BL/6 mice of 8–10 weeks (weighing 20–25 g) were used for animal experiments. Wild-type mice were purchased from Gempharmatech CO., Ltd (Nanjing, Jiangsu, China). Lcn2-/- mice (B6.129P2-Lcn2tm1Aade/AkiJ, Jax, 024630) were provided by the

	Jackson Laboratory.<br>Primary astrocytes were isolated from 1 to 2-day WT or Lcn2-/- pups ;<br>Primary microglia and oligodendrocyte were isolated from 1 to 2-day WT pups;<br>Primary cortical neurons were dissected from fetal WT C57BL/6 mice;<br>Primary brain microvascular endothelial cells were acquired from adult male WT C57BL/6 mice (6–8 weeks);<br>Myelin debris was isolated from adult male WT C57BL/6 mice brains (6–8 weeks).
Wild animals	This study did not use wild animals.
Field-collected samples	This study did not collect field samples
Ethics oversight	All experiments were carried out following the National Institutes of Health Guide for the Care and Use of Laboratory Animals, and were approved by the Animal Care Committee of Jinling Hospital.

Note that full information on the approval of the study protocol must also be provided in the manuscript.

Flow Cytometry

Plots

Confirm that:

- [x] The axis labels state the marker and fluorochrome used (e.g. CD4-FITC).
- [x] The axis scales are clearly visible. Include numbers along axes only for bottom left plot of group (a 'group' is an analysis of identical markers).
- [x] All plots are contour plots with outliers or pseudocolor plots.
- [x] A numerical value for number of cells or percentage (with statistics) is provided.

Methodology

Sample preparation	CFSE-labeled myelin debris was added to astrocytes for 24 hours. Myelin debris that was not ingested was washed away. Cells were collected and resuspended in PBS for immediate flow cytometry analysis with a BD FACS Canto flow cytometer.
Instrument	BD FACS Canto flow cytometer
Software	BD FACSDiva
Cell population abundance	We identified the percentage of GFAP and CFSE-myelin double-positive cells.
Gating strategy	Cells were firstly gated on FSC/SSC to exclude cell debris. Then CFSE-myelin positive cells were gated through FITC channel, we calculated the percentage of GFAP+ myelin+ double-positive cells.

- [x] Tick this box to confirm that a figure exemplifying the gating strategy is provided in the Supplementary Information.

Magnetic resonance imaging

Experimental design

Design type	Structural (T2WI) and diffusion (DWI and DTI) imaging.
Design specifications	T2WI and DWI were acquired to evaluate the infarct volume. DTI data was collected to assess the loss of myelin fibers
Behavioral performance measures	No behavioral tasks were performed.

Acquisition

Imaging type(s)	structural and diffusion
Field strength	7.0 T
Sequence & imaging parameters	T2-WI parameters: matrix = 256 × 256, field of view (FOV) = 20mm × 20mm, repetition time (TR) = 3000 ms, echo time (TE) = 36 ms, slice thickness = 0.6 mm.
Area of acquisition	Whole brain

Diffusion MRI [x] Used [ ] Not used

Parameters: DWI parameters: matrix = 128 × 128, FOV = 20mm × 20mm, TR = 5000 ms, TE = 30 ms, slice thickness = 0.6 mm;
DTI parameters: matrix = 128 × 128, FOV = 20mm × 20mm, TR = 5000 ms, TE = 32 ms, slice thickness = 0.6 mm.

Preprocessing

Preprocessing software	No preprocessing performed
Normalization	Not used.
Normalization template	No used.
Noise and artifact removal	Not used.
Volume censoring	Not used.

Statistical modeling & inference

Model type and settings	No modeling performed
Effect(s) tested	Not used.

Specify type of analysis: ☒ Whole brain ☐ ROI-based ☐ Both

Statistic type for inference (See Eklund et al. 2016)	Not relevant.
Correction	Not used.

Models & analysis

n/a	Involved in the study
☒	☐ Functional and/or effective connectivity
☐	☒ Graph analysis
☒	☐ Multivariate modeling or predictive analysis

Graph analysis	The infarct volume was calculated through ImageJ software according to DWI.
